# Systemic inflammation and B cell indices predict rituximab responses in membranous nephropathy

**DOI:** 10.1093/ckj/sfaf396

**Published:** 2025-12-18

**Authors:** Suyan Duan, Yuyou Ye, Qian Zhou, Hujia Hua, Ming Zeng, Chengning Zhang, Yanggang Yuan, Changying Xing, Huijuan Mao, Bo Zhang

**Affiliations:** Department of Nephrology, the First Affiliated Hospital of Nanjing Medical University, Nanjing Medical University, Nanjing, China; Department of Nephrology, the First Affiliated Hospital of Nanjing Medical University, Nanjing Medical University, Nanjing, China; Department of Nephrology, the First Affiliated Hospital of Nanjing Medical University, Nanjing Medical University, Nanjing, China; Department of Nephrology, the First Affiliated Hospital of Nanjing Medical University, Nanjing Medical University, Nanjing, China; Department of Nephrology, the First Affiliated Hospital of Nanjing Medical University, Nanjing Medical University, Nanjing, China; Department of Nephrology, the First Affiliated Hospital of Nanjing Medical University, Nanjing Medical University, Nanjing, China; Department of Nephrology, the First Affiliated Hospital of Nanjing Medical University, Nanjing Medical University, Nanjing, China; Department of Nephrology, the First Affiliated Hospital of Nanjing Medical University, Nanjing Medical University, Nanjing, China; Department of Nephrology, the First Affiliated Hospital of Nanjing Medical University, Nanjing Medical University, Nanjing, China; Department of Nephrology, the First Affiliated Hospital of Nanjing Medical University, Nanjing Medical University, Nanjing, China

**Keywords:** B lymphocyte, biomarkers, membranous nephropathy, rituximab, systemic inflammation response index

## Abstract

**Background:**

Membranous nephropathy (MN) is a frequent cause of nephrotic syndrome in adults with variable response to rituximab (RTX) therapy. While traditional markers like proteinuria and anti-phospholipase A2 receptor (PLA2R) antibodies exhibit predictive value, their limitations necessitate more robust biomarkers.

**Methods:**

We prospectively analysed 149 MN patients receiving RTX over 12 months. Inflammatory indices such as neutrophil:lymphocyte ratio (NLR), monocyte:lymphocyte ratio (MLR) and systemic inflammation response index (SIRI) together with B cell levels were measured alongside conventional markers at baseline, 3 months and 6 months. Predictive models for 6- and 12-month remission (complete/partial) were developed using multivariate regression and receiver operating characteristics (ROC) analysis.

**Results:**

Non-responders exhibited persistently elevated inflammatory markers (NLR, MLR, SIRI) throughout the entire observation period. Among the three, only SIRI can independently predict the remission of MN. At 3 months, SIRI ≤1.25 {odds ratio [OR] 3.68 [95% confidence interval (CI) 1.39–9.72]} and B cell proportion ≤0.2% [OR 2.90 (95% CI 1.00–8.35)] independently predicted 6-month response. Incorporating these two newly added indicators into the traditional variable model, which includes the levels of proteinuria, albumin and anti-PLA2R antibody at 3 months, markedly enhances prediction accuracy [area under the curve (AUC) 0.86 versus 0.81]. By 6 months, only SIRI ≤0.9 [OR 4.84 (95% CI 1.43–16.40)] and albumin change [OR 1.11 (95% CI 1.03–1.19)] predicted 12-month prognosis, as B cell and anti-PLA2R antibody levels lost significance. The prediction model incorporating SIRI also had better performance (AUC 0.82 versus 0.79).

**Conclusions:**

B lymphocyte levels constitute a robust predictive biomarker for assessing short-term therapeutic response in patients with MN receiving RTX therapy. Furthermore, SIRI emerges as a valuable prognostic indicator capable of predicting both short-term efficacy and long-term renal outcomes. These findings suggest that concurrent monitoring of B lymphocyte levels and SIRI values warrants integration into standardized monitoring frameworks within clinical management protocols.

## Graphical Abstract

**Graphical Abstract:**
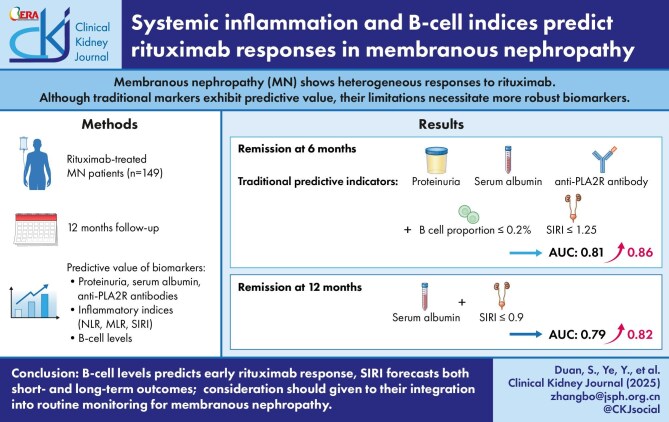

KEY LEARNING POINTS **What was known:**

- Traditional predictors—proteinuria, serum albumin and anti-phospholipase A2 receptor (PLA2R) antibodies—offer limited and delayed prognostic accuracy for rituximab response in membranous nephropathy (MN). However, easily accessible inflammatory–immune composite biomarkers had been prospectively validated for early or long-term outcome prediction.

**This study adds:**

- We demonstrated that at 3 months SIRI ≤1.25 and a peripheral B cell level ≤0.2% independently predicted 6-month remission, improving the area under the curve from 0.81 to 0.86.
- By 6 months, only SIRI ≤0.9 and the albumin increment remained predictive of 12-month outcomes, whereas anti-PLA2R titres and B cell levels lost significance.

**Potential impact:**

- Incorporating SIRI and B cell monitoring into routine clinical surveillance could enable earlier treatment response stratification and individualized rituximab adjustment, potentially reduce overtreatment and enhance long-term renal prognosis in patients with MN.

## INTRODUCTION

Membranous nephropathy (MN) is a glomerular disease affecting individuals across all age groups and represents the most common cause of nephrotic syndrome in adults [1, 2]. Management of MN includes both supportive therapies and immunosuppressive interventions. Rituximab (RTX), a monoclonal antibody targeting CD20, is currently employed as the first-line

immunosuppressive agent. By inducing B cell depletion, RTX reduces autoantibody production, thereby mitigating immune-mediated kidney injury [3]. Although the overall response rate to RTX in the treatment of MN ranges between 60% and 80%, patient responses vary considerably both in the early phase and over the long term. Traditional indicators used to predict the efficacy of RTX include proteinuria level, serum albumin concentration and the urine protein:creatinine ratio (UPCR) [4]. Moreover, anti-phospholipase A2 receptor (PLA2R) antibody levels have been validated as early markers for predicting RTX efficacy [5]. However, inconsistencies between antibody titres and treatment response, along with delays in titre changes, limit their effectiveness as prognostic tools.

As research deepens, numerous novel biomarkers have been identified. For instance, anti-semaphorin 3B antibodies exhibit a strong correlation with disease activity [6], whereas anti-HTRA1 antibody titres correlate with disease duration and decrease upon disease resolution [7]. The identification of these novel markers offers additional guidance for the administration of RTX treatment. However, acquiring such data remains challenging because these tests are not yet widely implemented in clinical practice.

Given the intrinsic inflammatory–immune imbalance observed in patients with MN, the present study examined a series of readily accessible composite inflammatory indices, including the neutrophil:lymphocyte ratio (NLR), monocyte:lymphocyte ratio (MLR), systemic inflammatory response index (SIRI) and immune parameters such as B lymphocyte levels. These biomarkers have demonstrated significant clinical utility across a range of conditions, including various cancers, rheumatoid arthritis and COVID-19, by effectively reflecting alterations in both inflammatory status and immune function [8–11, 12]. Among them, SIRI is a novel biomarker and an integrative haematological metric that amalgamates circulating neutrophil, monocyte and lymphocyte counts to provide a dynamic portrait of both innate and adaptive immune signals [13]. An elevated SIRI level reflects a state of amplified systemic inflammation coupled with concomitant immunosuppression [14]. Qin *et al*. [15] previously demonstrated a positive correlation between elevated SIRI and increased proteinuria excretion. However, their application in nephropathy, and MN in particular, remains underexplored. This study aims to investigate and validate the role of these novel biomarkers, in conjunction with traditional markers, for predicting both the early and long-term efficacy and prognosis of RTX treatment in patients with MN. Ultimately, our goal is to establish a more precise foundation for clinical decision-making, thereby enhancing treatment outcomes and patient prognoses.

## MATERIALS AND METHODS

### Patients

**Figure 1:**
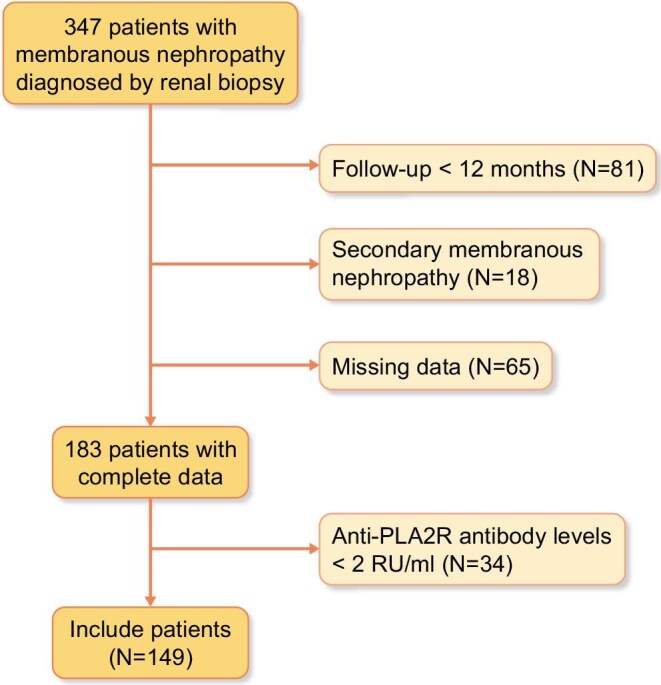
Flow chart of participant selection.

Patients diagnosed with MN via renal biopsy at our hospital or external institutions who subsequently underwent RTX treatment at our hospital from 2014 to 2023 were included in this study. The baseline was established as the time of their initial RTX administration at our hospital. All patients had baseline anti-PLA2R antibody levels ≥2 RU/ml. Patients with secondary MN and those with significant loss of clinical data were excluded. The patient enrolment process is shown in Fig. 1. In light of the individual circumstances of the patients, they will undergo one of the several treatment regimens as follows:

- 375 mg/m^2^ protocol: RTX is administered intravenously at a dose of 375 mg/m^2^ each time, once a week for 2–4 consecutive weeks [16].
- B cell level–driven protocol: The initial dose of RTX is calculated based on body surface area, with a minimum of 150 mg/m^2^. The final dose is determined as the higher value in multiples of 100 mg. The first follow-up is carried out after 2 weeks and the circulating B cells are measured every 2 months thereafter. During the follow-up, if the B cell count is >5/mm^3^, 100 mg, 75 mg/m^2^ or 150 mg/m^2^ is administered based on the change in PLA2R antibody. If the B cell count is <5/mm^3^, only 100 mg or 75 mg/m^2^ is given when the PLA2R antibody remains unchanged or increases; otherwise, no additional treatment is provided [17].
- Other low-dose protocol: brief fixed-dose regimens (e.g. 500 mg on days 1 and 8) [16] or monthly mini-dose maintenance (100 mg every 4 weeks until anti-PLA2R seronegativity or proteinuria remission) [18].

At the 6-month follow-up, patients were re-evaluated. A second course of RTX was administered only if there was a ≥25% decrease in proteinuria from baseline, yet a complete response (CR) had not been achieved by 6 months. This decision-making was independent of CD19-positive B cells. Patients who achieved a complete response at 6 months did not receive additional doses. And those with a <25% reduction in proteinuria were considered non-responders and were not given further doses of RTX [19].

This study was approved by the Ethics Committee of the First Affiliated Hospital of Nanjing Medical University (approval no. 2024-SR-431) and conducted in accordance with the principles of the Declaration of Helsinki. All individual participants in the study signed written informed consent forms.

### Data acquisition

Baseline assessments included the collection of demographic information, clinical symptoms, clinical trial data and renal pathological biopsy results. Subsequently, clinical parameters such as serum creatinine, albumin, 24-h urinary protein, anti-PLA2R antibody levels and B lymphocyte levels (specifically referring to the percentage of CD19-positive B lymphocytes). The above indices were assessed synchronously at 3, 6, 9 and 12 months after treatment. The primary endpoints were defined as 12 months of CR (proteinuria <0.3 g/day and albumin ≥35 g/l) and partial response (PR) as a 50% reduction in albuminuria from baseline and ≤3.5 g/day.

### Assessment of exposed variables

NLR [20] was first proposed by Zahorec in 2001 [21]. Through the simple ratio of neutrophils (pro-inflammatory) to lymphocytes (anti-inflammatory), it instantly and quantitatively reflects the body’s systemic inflammatory state. In addition, it has been shown to correlate positively with the degree of inflammation and poor prognosis in a variety of diseases, including infections, cardiovascular events, tumours and autoimmune disorders [22, 23].

MLR [24] reflects the balance between monocyte and lymphocyte levels and reflects the circulating immune status of the body [25]. Its significant impact on the progression of nephritis and treatment outcomes in various solid tumours has been conclusively validated [26, 27].

SIRI [(neutrophil count*monocyte count)/lymphocyte count] [28], first described by Qi *et al.* in 2016 [29], has emerged as a comprehensive and innovative inflammation biomarker based on immune cell subpopulations for assessing systemic inflammatory status and immune response [30]. Accumulating evidence has established SIRI as a robust prognostic indicator where elevated values are closely associated with cardiovascular diseases and mortality, adverse cancer outcomes and aggravation of infectious diseases [31–34].

### Statistical analysis

Descriptive analyses were conducted at three critical time points: baseline, 3 months and 6 months. For quantitative variables with a normal distribution, the data were characterized using mean ± standard deviation (SD) and intergroup comparisons were performed using the independent samples Student’s *t*-test. Quantitative variables that did not follow a normal distribution were described by the median and interquartile range (IQR), with the Mann–Whitney U test employed for between-group comparisons. Qualitative variables were compared between groups using the chi-squared test. When necessary, through receiver operating characteristic (ROC) curve analysis, the Youden index was calculated to determine the optimal cut-off value of the continuous variable, so as to realize the optimal classification of the variable. The best parameterization for each continuous variable was based on model fit. At 3 and 6 months, univariate logistic regression analysis was conducted to identify clinical predictors associated with the outcome (*P* < .05). Subsequently, multivariate logistic regression analysis was performed for further refinement. Both traditional and novel prediction models were established and ROC curves were generated to compare their abilities to predict disease remission.

## RESULTS

### Patient features

Based on the remission status at 12 months, patients were stratified into two groups: those who achieved remission and those who did not. The remission group consisted of 105 patients, 53 of whom achieved PR and 52 who achieved CR, while the non-remission group comprised 44 patients. At baseline, no significant differences were observed between the two groups regarding sex, age or BMI. Prior immunosuppression was defined as the previous use of glucocorticoids, cyclophosphamide or calcineurin inhibitors. In the non-remission group, 35 patients (79.5%) had a history of prior immunosuppression, compared with 77 patients (73.3%) in the remission group. No statistically significant differences were observed between the two groups in this regard (Table 1). Serum albumin levels were significantly lower in patients who did not achieve remission compared with those who did [median 19.55 g/dl (IQR 16.85–24.80) versus 23.70 (19.30–28.30), *P* = .005] (Table 1). In addition, the 24-h proteinuria level in the non-remission group was significantly higher than that in the remission group [median 8.64 g/day (IQR 5.06–13.80) versus 5.41 (3.38–8.20), *P* < .001] (Table 1). Creatinine levels were significantly elevated in the non-remission group compared with the remission group [median 82.10 μmol/l (IQR 60.00–112.90) versus 73.00 (61.10–85.70), *P* = .022] (Table 1). Anti-PLA2R antibody levels in the non-remission group were significantly higher than those in the remission group [median 125.62 RU/ml (IQR 58.31–308.63) versus 73.10 (21.01–222.61), *P* = .021] (Table 1). At baseline, the non-remitted group exhibited a significantly elevated NLR compared with the remitted group [median 2.57 (IQR 1.85–3.25) versus 2.09 (1.55–2.83), *P* = .021] (Table 1) and significantly higher levels of SIRI [median 1.38 (IQR 0.90–2.07) versus 0.87 (0.64–1.44), *P* = .004] (Table 1). The MLR in the non-remission group was higher than that in the remission group; however, the difference between the two groups did not reach statistical significance [median 0.27 (IQR 0.22–0.37) versus 0.24 (0.19–0.34), *P* = .091] (Table 1). The non-remission group exhibited a higher B cell proportion than the remission group; however, this difference did not reach statistical significance (14.77 ± 6.29 versus 13.13 ± 6.86%, *P* = .176) (Table 1). The choice of regimen did not differ statistically between the two groups. We tallied the total dose of RTX administered to the enrolled patients over the 6-month treatment period and found that the total dose was significantly higher in the non-remission group than in the remission group [median 1100 mg (IQR 800–1400) versus 800 (600–1200), *P* = .006] (Table 1). We further calculated the ratio of the 6-month total dose to patient body weight and found no significant difference between the two groups [median 15.66 mg/kg (IQR 11.05–19.82) versus 13.65 (9.26–17.65), *P* = .099] (Table 1).

**Table 1:** Baseline clinical characteristics of the patients.

Baseline characteristics	Overall cohort	No remission at 12 months	Remission at 12 months	P-value
Patients, n	149	44	105	
Age (years)	54.2	54 (44.5–61.0)	58 (42.5–67)	.295
Male, n (%)	88 (59.1)	29 (66.0)	59 (56.2)	.271
BMI (kg/m2)	23.38 (21.62–25.28)	23.51 (21.32–27.80)	23.42 (21.70–24.60)	.314
Prior immunosuppression, n (%)	112(75.2)	35(79.5)	77(73.3)	.423
Albumin (g/dl)	22.90 (17.80–27.30)	19.55 (16.85–24.80)	23.70 (19.30–28.30)	.005
Proteinuria (g/day)	6.49 (3.54–9.49)	8.64 (5.06–13.80)	5.41 (3.38–8.20)	<.001
Serum creatinine (μmol/l)	73.90 (61.10–90.10)	82.10 (60.00–112.90)	73.00 (61.10–85.70)	.022
Anti-PLA2R antibody level (RU/ml)	90.77 (35.61–234.50)	125.62 (58.31–308.63)	73.10 (21.01–222.61)	.021
NLR	2.20 (1.63–2.96)	2.57 (1.85–3.25)	2.09 (1.55–2.83)	.021
MLR	0.25 (0.20–0.35)	0.27 (0.22–0.37)	0.24 (0.19–0.34)	.091
SIRI	1.02 (0.69–1.83)	1.38 (0.90–2.07)	0.87 (0.64–1.44)	.004
FAR	0.18 (0.12–0.24)	0.22 (0.15–0.31)	0.17 (0.11–0.23)	.004
Non-HDL (mmol/l)	5.56 (4.06–7.35)	6.14 (4.57–7.80)	5.34 (4.02–7.11)	.075
Mean arterial pressure (mmHg)	95.00 (85.67–105.38)	97.50 (90.50–105.33)	94.33 (88.67–106.00)	.566
B lymphocyte proportion (%), mean ± SD	13.62 ± 6.72	14.77 ± 6.29	13.13 ± 6.86	.176
Therapeutic protocol, n (%)				.365
375 mg/m2	34 (22.8)	7 (15.9)	27 (25.7)	
B cell level–driven	105 (70.5)	33 (75)	72 (68.6)	
Other low-dose	10 (6.7)	4 (9.1)	6 (5.7)	
Total dose of RTX within 6 months (mg)	900 (600–1200)	1100 (800–1400)	800 (600–1200)	.006
Dose:body weight ratio (mg/kg)	13.79 (9.52–18.71)	15.66 (11.05–19.82)	13.65 (9.26–17.65)	.099

Values are presented as median (IQR) unless stated otherwise.

Prior immunosuppression, including prior treatment with cyclosporine, tacrolimus or other immunosuppressive agents.

FAR: fibrinogen:albumin ratio; non-HDL (non-high-density lipoprotein cholesterol) = total cholesterol − high-density lipoprotein cholesterol.

Mean arterial pressure = diastolic blood pressure + (systolic blood pressure = diastolic blood pressure)/3.

### Clinical parameter trends

**Figure 2:**
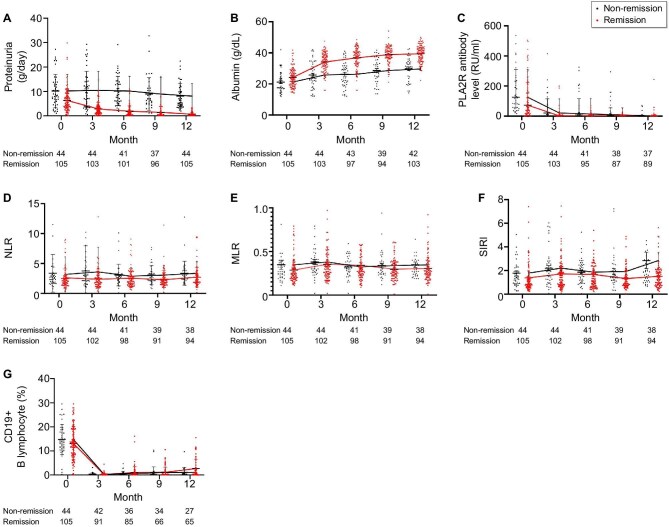
Clinical indicator changes based on the remission status at 12 months. **(A)** Proteinuria levels over time. **(B)** Albumin levels over time. **(C)** Anti-PLA2R antibody levels over time. **(D)** NLR levels over time. **(E)** MLR over time. **(F)** SIRI levels over time. **(G)** CD19-positive B lymphocyte levels over time.

Fig. 2 illustrates the trajectories of key clinical measures from baseline to 12 months of follow-up in both the remission and non-remission groups. At baseline, anti-PLA2R antibody levels were significantly higher in the non-remission group than in the remission group. At 3 months, both groups experienced a marked decline in anti-PLA2R antibody levels, which then stabilized and was maintained at lower values (Fig. 2C). Over the 12-month follow-up period, the trends in NLR, MLR and SIRI were generally elevated in the non-remission group relative to the remission group (Fig. 2D–F). In contrast, the levels and trends of B lymphocytes remained essentially consistent between the two groups (Fig. 2G).

### Short-term predictors of RTX-induced remission in MN at 6 months

Table 2 details the clinical characteristics of patients in both the non-remission and remission groups. In addition to differences in the absolute values of relevant indicators between the two groups, significant disparities were observed in the magnitude of changes in these indicators over the 3-month period, reflecting differential rates of renal function recovery. At the 3-month time point, we conducted an in-depth analysis to determine whether integrating clinical parameters—specifically B lymphocyte levels and a panel of inflammatory composite markers—with traditional markers of MN could enhance the prediction of disease remission at 6 months. Univariate analysis demonstrated that at 3 months a SIRI ≤1.25 {odds ratio [OR] 2.26 [95% confidence interval (CI) 1.12–4.54], *P* = .022}, anti-PLA2R antibody levels ≤10 RU/ml [OR 6.76 (95% CI 3.14–14.53), *P* < .001) and B lymphocyte proportion ≤0.2% [OR 4.43 (95% CI 1.94–10.11), *P* < .001] were significantly associated with disease remission at 6 months. Additionally, the analysis revealed that reductions in proteinuria [OR 1.00 (95% CI 1.00–1.00), *P* = .001] and elevations in albumin [OR 1.16 (95% CI 1.09–1.24), *P* < .001] levels were significantly correlated with disease remission. However, the choice of different RTX treatment protocols had no significant impact on patient outcomes at 6 months (*P* = .306); after adjustment for demographics, comorbidities and concomitant medications, the results remained non-significant (Supplementary Table 1).

**Table 2:** Clinical characteristics of the patients at 3 months of follow-up.

3-month characteristics	No remission at 12 months	Remission at 12 months	*P*-value
Serum creatinine (μmol/l)	81.30 (66.25–117.50)	74.20 (63.70–88.70)	.064
Anti-PLA2R antibody level (RU/ml)	11.90 (1.97–108.34)	2.26 (1.63–7.50)	<.001
Anti-PLA2R antibody level change from baseline to 3 months (per RU/ml decrease)	−78.82 (−183.94 to −30.12)	−64.32 (−216.90 to −16.41)	.821
Proteinuria (g/day)	9.42 (3.81–13.34)	2.35 (1.15–3.79)	<.001
Proteinuria change from baseline to 3 months (per g/day decrease)	1.71 (−4.32–2.97)	−2.40 (−4.74 to −0.10)	<.001
Albumin (g/dl), mean ± SD	25.76 ± 8.22	34.10 ± 6.04	<.001
Albumin change from baseline to 3 months (per g/l increase)	3.95 (−0.10–8.20)	9.20 (5.60–14.30)	<.001
B lymphocyte proportion (%)	0.15 (0.10–0.40)	0.10 (0.00–0.20)	.006
B lymphocyte proportion change from baseline to 3 months (per % decrease)	−14.00 (−18.10 to −10.90)	−12.50 (−19.00 to −7.90)	.141
NLR	2.74 (1.88–3.83)	2.15 (1.47–3.05)	.023
NLR change from baseline to 3 months	0.06 (−0.36–1.16)	0.05 (−0.46–0.48)	.392
MLR	0.35 (0.27–0.42)	0.31 (0.24–0.41)	.112
MLR change from baseline to 3 months	0.08 (−0.01–0.14)	0.06 (−0.00–0.13)	.671
SIRI	1.98 (1.20–2.61)	1.31 (0.77–2.32)	.014
SIRI change from baseline to 3 months	0.31 (−0.09–0.99)	0.27 (−0.08–0.78)	.358

Values presented as median (IQR).

Incorporating these factors into a multivariate analysis, the results revealed that changes in albumin levels, SIRI, anti-PLA2R antibody levels and B lymphocyte levels are independent predictors of disease remission at 6 months. Specifically, each 1 g/l increase in albumin levels from baseline to 3 months was associated with a significantly higher likelihood of achieving remission at 6 months [OR 1.11 (95% CI 1.02–1.20), *P* = .013]. At 3 months, a SIRI value ≤1.25 was associated with a remission rate that increased by >3-fold [OR 3.68 (95% CI 1.39–9.72), *P* = .009]. Anti-PLA2R antibody levels ≤10 were significantly associated with a remission rate more than five times higher than that observed in patients with elevated levels [OR 5.22 (95% CI 2.00–13.54), *P* < .001]. Of note, a B lymphocyte proportion ≤0.2% at 3 months was a reliable predictor of response, which increased the likelihood of response nearly 3-fold [OR 2.90 (95% CI 1.00–8.35), *P* = .049] (Table 3).

**Table 3:** Univariate and multivariate logistic regression models for MN patients in remission after 6 months of RTX treatment.

	Univariable or bivariable models*		Multivariable model	
Factors	OR (95% CI)	*P*-value	OR (95% CI)	*P*-value
Serum creatinine change from baseline to 3 months (per μmol/l decrease)	1.00 (1.00–1.01)	.392		
Proteinuria change from baseline to 3 months (per g/day decrease)	1.00 (1.00–1.00)	.001	1.00 (1.00–1.00)	.298
Albumin change from baseline to 3 months (per g/l increase)	1.16 (1.09–1.24)	<.001	1.11 (1.02–1.20)	.013
NLR ≤3 at 3 months	1.59 (0.79–3.22)	.195		
MLR ≤0.25 at 3 months	1.38 (0.65–2.92)	.405		
SIRI ≤1.25 at 3 months	2.26 (1.12–4.54)	.022	3.68 (1.39–9.72)	.009
Anti-PLA2R antibody level ≤10 at 3 months (RU/ml)	6.76 (3.14–14.53)	<.001	5.22 (2.00–13.54)	<.001
B lymphocyte proportion ≤0.2 at 3 months (%)	4.43 (1.94–10.11)	<.001	2.90 (1.00–8.35)	.049
Treatment protocol	1.43 (0.72–2.83)	.306		
375 mg/m^2^	1.00 (Reference)			
B cell level–driven	1.29 (0.58–2.84)	.533		
Other low-dose	0.70 (0.17–2.88)	.621		

**Figure 3:**
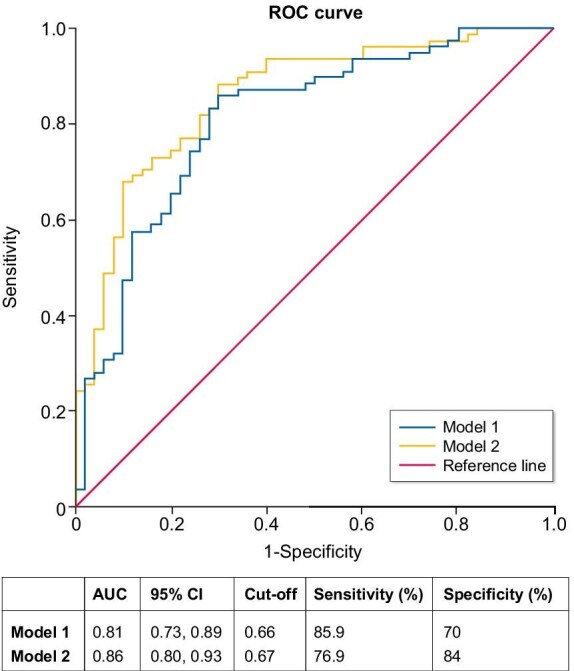
Predictive model for response in patients with MN at 6 months of RTX therapy (ROC curve). Model 1: proteinuria change from baseline to 3 months + albumin change from baseline to 3 months + anti-PLA2R antibody levels at 3 months. Model 2: proteinuria change from baseline to 3 months + albumin change from baseline to 3 months + anti-PLA2R antibody levels at 3 months + SIRI at 3 months + CD19-positive B lymphocyte levels at 3 months.

Subsequently we developed two logistic regression models and conducted an ROC curve analysis to evaluate the predictive accuracy of 6-month remission in patients with MN treated with RTX. Model 1 includes a proteinuria change from baseline to 3 months, albumin change from baseline to 3 months and anti-PLA2R antibody levels at 3 months. Model 1 demonstrated an AUC of 0.81 (95% CI 0.73–0.89), with a sensitivity of 85.9% and a specificity of 70% at an optimal cut-off value of 0.66 (Fig. 3). In contrast, model 2, which integrated SIRI and B lymphocyte levels, achieved an AUC of 0.86 (95% CI 0.80–0.93), with a sensitivity of 76.9% and a specificity of 84% at a cut-off value of 0.67 (Fig. 3). The ROC curve analysis indicated that model 2, by incorporating a broader range of clinical parameters, exhibited superior predictive performance compared with model 1.

### Long-term predictors of RTX-induced remission in MN at 12 months

Table 4 presents clinical indicators for patients at the 6-month follow-up. Consistent with the 3-month follow-up data, significant intergroup differences were observed not only in proteinuria and serum albumin levels but also in the magnitude of changes in these parameters between the non-remission and remission groups (*P* < .001). Serum creatinine levels were significantly elevated in the non-remission group compared with the remission group (*P* = .018). Additionally, the non-remission group exhibited significantly elevated NLRs (*P* = 0.008) and SIRIs (*P* = .020) compared with the remission group. Similarly, univariate and multivariate logistic regression analyses were performed to identify predictors of 12-month remission among patients with RTX-induced MN. In the univariate analysis, remission at 12 months was significantly associated with reduced proteinuria [OR 1.00 (95% CI 1.00–1.00), *P* < .001] and elevated albumin levels [OR 1.16 (95% CI 1.09–1.23), *P* < .001]. Additionally, an NLR of ≤3 [OR 3.50 (95% CI 1.47–8.35), *P* = .005] and a SIRI of ≤0.9 at 6 months [OR 3.39 (95% CI 1.30–8.83), *P* = .013] were predictive of remission. The PLA2R antibody level of ≤2 RU/ml at 6 months was associated with remission [OR 4.47 (95% CI 2.00–10.00), *P* < .001]. The B lymphocyte proportion at 6 months was not significantly associated with remission at 12 months [OR 1.19 (95% CI 0.54–2.61), *P* = .670]. Throughout the 12-month observation period, no differences in long-term prognosis were observed among the treatment-regimen groups (*P* = .346). Additionally, in line with the short-term efficacy results, the association remained non-significant after additional adjustment for potential confounders (Supplementary Table 2). Furthermore, in the multivariate analysis, both albumin change [OR 1.11 (95% CI 1.03–1.19), *P* = .006] and lower SIRI [OR 4.84 (95% CI 1.43–16.40), *P* = .011] at 6 months were independently associated with remission. At 6 months, the anti-PLA2R antibody titre [OR 2.44 (95% CI 0.90–6.61), *P* = .079] did not retain an independent predictive value in the multivariate analysis (Table 5).

**Table 4:** Clinical characteristics of the patients at 6 months of follow-up.

6 months characteristics	No remission at 12 months	Remission at 12 months	*P*-value
Serum creatinine (μmol/l)	79.75 (65.05–108.60)	74.60 (65.30–90.10)	.018
Anti-PLA2R antibody level (RU/ml)	10.55 (2.04–117.00)	2.00 (1.68–11.70)	<.001
Anti-PLA2R antibody level change from baseline to 6 months (per RU/ml decrease)	−75.62 (−193.33 to −30.12)	−84.78 (−232.42 to −23.80)	.539
Proteinuria (g/day)	9.20 (5.17–14.34)	1.42 (0.58–2.66)	<.001
Proteinuria change from baseline to 6 months (per g/day decrease)	0.07 (−5.35–3.84)	−3.43 (−6.06 to −1.57)	<.001
Albumin (g/l), mean ± SD	25.71 ± 8.71	36.01 ± 6.73	<.001
Albumin change from baseline to 6 months (per g/l increase), mean ± SD	5.03 ± 6.79	12.94 ± 7.45	<.001
B lymphocyte proportion (%)	0.30 (0.10–0.70)	0.30 (0.10–0.90)	.603
B lymphocyte proportion change from baseline to 6 months (per % decrease), mean ± SD	−13.55 ± 6.56	−12.13 ± 7.24	.312
NLR	2.78 (2.22–3.23)	2.01 (1.45–2.87)	.008
NLR change from baseline to 6 months	0.32 (−0.12–0.86)	0.07 (−0.52–0.70)	.268
MLR	0.31 (0.25–0.38)	0.27 (0.22–0.36)	.183
MLR change from baseline to 6 months	0.05 (−0.03–0.10)	0.03 (−0.05–0.10)	.904
SIRI	1.66 (1.01–2.58)	1.13 (0.66–2.14)	.020
SIRI change from baseline to 6 months	0.37 (−0.32–1.08)	0.15 (−0.16–0.67)	.486

Values presented as median (IQR) unless stated otherwise.

**Table 5:** Univariate and multivariate logistic regression models for MN patients in remission after 12 months of RTX treatment.

	Univariable or bivariable models*		Multivariable model	
Factors	OR (95% CI)	*P*-value	OR (95% CI)	*P*-value
Serum creatinine change from baseline to 6 months (per μmol/l decrease)	1.00 (1.00–1.01)	.320		
Proteinuria change from baseline to 6 months (per g/day decrease)	1.00 (1.00–1.00)	<.001	1.00 (1.00–1.00)	.104
Albumin change from baseline to 6 months (per g/l increase)	1.16 (1.09–1.23)	<.001	1.11 (1.03–1.19)	.006
NLR ≤3 at 6 months	3.50 (1.47–8.35)	.005	1.65 (0.60–4.54)	.329
MLR ≤0.25 at 6 months	1.98 (0.85–4.61)	.115		
SIRI ≤0.9 at 6 months	3.39 (1.30–8.83)	.013	4.84 (1.43–16.40)	.011
Anti-PLA2R antibody level ≤2 at 6 months (RU/ml)	4.47 (2.00–10.00)	<.001	2.44 (0.90–6.61)	.079
B lymphocyte proportion ≤0.2 at 6 months (%)	1.19 (0.54–2.61)	.670		
Treatment protocol	1.78 (0.30–3.95)	.346		
375 mg/m^2^	1.00 (Reference)			
B cell level–driven	0.82 (0.34–1.96)	.821		
Other low-dose	0.84 (0.18–3.97)	.840		

**Figure 4:**
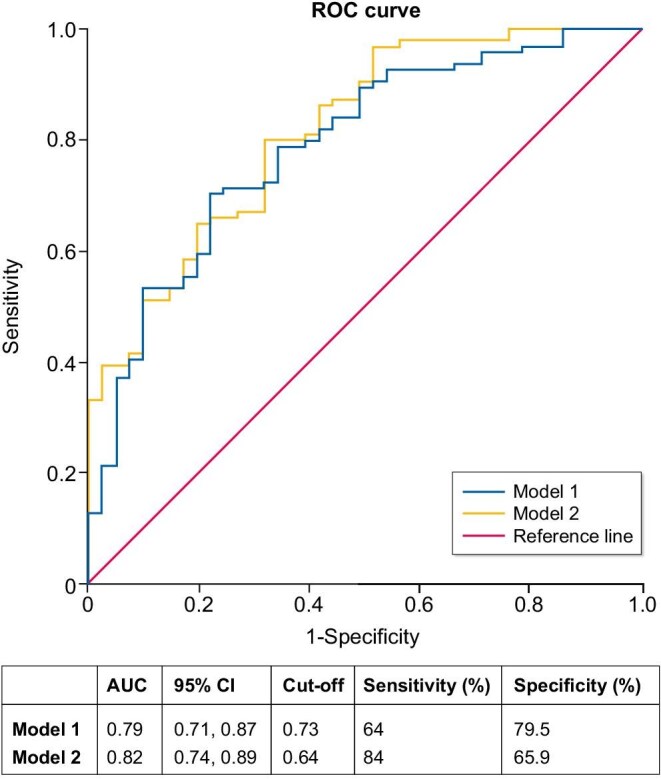
Predictive model for response in patients with MN at 12 months of RTX therapy (ROC curve). Model 1: proteinuria change from baseline to 6 months + albumin change from baseline to 6 months. Model 2: proteinuria change from baseline to 6 months + albumin change from baseline to 6 months + SIRI at 6 months.

At the 6-month time point, two logistic regression models were developed and their predictive accuracies for 12-month remission in patients with MN treated with RTX were evaluated using ROC curve analysis (Fig. 4). Model 1 incorporated changes in proteinuria and albumin levels at 6 months, yielding an AUC of 0.79 (95% CI 0.71–0.87). With a cut-off value of 0.73, its sensitivity and specificity were 64% and 79.5%, respectively. In contrast, model 2, which additionally incorporated the SIRI alongside the changes in proteinuria and albumin levels, demonstrated improved performance with an AUC of 0.82 (95% CI 0.74–0.89). At a cut-off value of 0.64, model 2 exhibited a sensitivity of 84% and a specificity of 65.9%.

## DISCUSSION

Our study introduced several representative inflammatory composite indices, including the NLR, MLR, SIRI and B lymphocyte levels, which reflects aspects of immune capacity. A cohort of 149 patients with MN treated with RTX was followed for 12 months, with clinical and laboratory parameters analysed every 3 months, focusing primarily on the baseline, 3-month and 6-month assessments. The findings revealed that both non-responders and responders exhibited sustained differences in inflammatory status and immune profiles throughout the follow-up period. Notably, when these inflammatory and immune indices were combined with traditional predictive factors (e.g. serum albumin, proteinuria), they demonstrated superior predictive value for both short-term (3–6 months) and long-term (12 months) treatment outcomes compared with conventional biomarkers alone.

Examining the longitudinal course of disease treatment, we identified several critical time points. First, the treatment response peaked at 3 months. In the responder group, proteinuria decreased rapidly while serum albumin levels increased, establishing a clear distinction compared with the non-remission group. At the 3-month mark, significant differences between the two groups were observed in both proteinuria and albumin levels, whether assessed through absolute values or changes over the period. These findings are consistent with those reported by Barbour *et al.* [35]. These findings indicate that proteinuria and serum albumin not only serve as continuous predictors of treatment response, but also that more rapid decreases in proteinuria and quicker recovery of albumin levels are frequently associated with a higher likelihood of eventual remission following treatment. During this period, the non-remission group exhibited elevated levels of NLR and SIRI, as well as a higher proportion of B lymphocytes. Combining the results of logistic regression, we found that 3 months after RTX treatment, employing SIRI and B lymphocyte levels in conjunction with proteinuria, albumin and PLA2R antibody levels—factors previously demonstrated to be strongly associated with disease prognosis [36–38]—yielded a higher predictive value than the traditional indicators used in isolation. At the 6-month follow-up, significant differences in proteinuria and albumin levels, as well as their respective changes, were observed between the two groups, thereby confirming our earlier hypothesis regarding prognostic significance of the rate of change across both early and late treatment stages. Moreover, during this period the non-remission group continued to exhibit relatively active inflammatory responses, as evidenced by sustained elevations in NLR and SIRI into the later stages of treatment.

At the 6-month time point, the predictive value of PLA2R antibody and B lymphocyte levels diminished, which is consistent with previous findings [35]. From an immunological standpoint, short-term prognosis is less influenced by residual antibodies, whereas the prolonged disease course permits the gradual emergence of effects from long-lived plasma cells (LLPCs). Specifically, although antibodies produced by short-lived plasma cells are largely eliminated after 6 months, LLPCs may persist in secreting low-level antibodies that are resistant to clearance by RTX, resulting in to a deceleration or plateau in the decline of antibody levels [39]. At this juncture, the association between antibody titres and clinical remission becomes attenuated. Previous studies found that B cell depletion was characterized by an immediate effect, with a profound depletion observed as early as 3 months [40]. Typically, B cells begin to repopulate within 6–12 months, but the reconstitution patterns vary among different B cell subgroups. Thus, further differentiation of B cell subsets may be necessary to enhance predictive accuracy. Moreover, the maintenance of immune homeostasis associated with a reduced total B cell level appears to be more beneficial in the early stages of treatment [41]. Furthermore, from a pathophysiological perspective, antibody synthesis is interrupted within weeks to months, but the structural consequences already imprinted on the glomerulus resolve more slowly [42]. Accordingly, treatment-remission biopsies show vanishing immune deposits and glomerular basement membrane (GBM) ‘holes’, signifying immune-complex clearance and partial restoration of GBM integrity [43]. Consequently, proteinuria continues to decrease for 3–6 months after seroconversion, creating an ‘immunostructural time window’ during which antibody levels no longer reflect active injury. During this lag, immune complement–mediated podocyte damage may be resolved through other mechanisms, e.g. mesangial and infiltrating macrophage clearance of residual deposits [40]. The finding that a lower 6-month SIRI conferred superior predictive power for 12-month remission indicates that modulating the pro-inflammatory versus immunosuppressive balance during this critical window is a key determinant of long-term disease control. Finally, although NLR and MLR did not exhibit high predictive value in our study, prior research has identified an elevated NLR as an independent risk factor for MN [44]. Analysis of these markers during follow-up revealed that the non-remission group consistently exhibited higher inflammatory activity compared with the remission group. Notably, at 6 months, the overlapping values of NLR, MLR and SIRI between the two groups may signify an intermediate phase of immune and inflammatory regulation, potentially serving as a critical juncture that distinguishes between disease recurrence and remission.

Our findings are of considerable clinical importance. The hallmark pathological alterations observed in MN arise from B cell–mediated autoantibody production (e.g. anti-PLA2R antibodies) combined with sustained complement system activation, ultimately resulting in immune complex deposition on podocyte surfaces and subsequent local inflammatory responses [45, 46]. Thus disease progression is influenced not only by the clearance of autoantibodies but also significantly influenced by the systemic inflammatory state and the patterns of B cell depletion and reconstitution. Traditional predictive models tend to exhibit a delay, making them inadequate for promptly capturing immunological changes and the low-grade inflammation that may persist even after antibody negativity, ultimately affecting long-term prognosis. The SIRI is a composite indicator that reflects the balance among neutrophils, monocytes and lymphocytes, thereby capturing early alterations in the immune microenvironment during treatment and facilitating the early identification of responders. In the later stages of treatment, due to its downstream role in the pathogenic cascade, SIRI exhibits a prolonged effect, providing a more reliable reflection of long-term prognosis (≥12 months). In contrast, B cell monitoring primarily predicts significance for short-term treatment responses, a relationship closely tied to the immunological characteristics of MN. RTX rapidly reduces autoantibody production through the depletion of clearing CD20-positive B cells, thus the extent of early B cell depletion, which represents an upstream event in disease progression, serves as a more effective and earlier predictor of short-term efficacy. Accordingly, clinical practice should incorporate SIRI as a routine monitoring indicator during RTX treatment for MN to enhance the accuracy of efficacy assessments and support individualized treatment adjustments. Future investigations should further explore the prognostic value of various inflammatory indicators (e.g. platelet:lymphocyte ratio) and specific immune cell subsets (e.g. T follicular helper cells, regulatory B cells) [47] and cytokines (e.g. B cell activating factor of the tumour necrosis factor family/a proliferation-inducing ligand) within this predictive model to optimize long-term management strategies for MN patients.

## Supplementary Material

sfaf396_Supplemental_File

## Data Availability

The datasets used and/or analysed during the current study are available from the corresponding author upon reasonable request.
