# Supplementary material for: Systemic inflammation and B cell indices predict rituximab responses in membranous nephropathy
Source: Clin Kidney J. 2025 Dec 18;19(2):sfaf396. doi: 10.1093/ckj/sfaf396 (PMC12873549; doi:10.1093/ckj/sfaf396)
Supplement: sfaf396_Supplemental_File [file sfaf396_supplemental_file.docx]

**Supplementary Table 1 Effects of different rituximab regimens on 6-month outcomes (crude to multivariable-adjusted analyses)**

| \| Variables \| Remission (%) \| P \| Model1 \| \|  \| Model2 \| \|  \| Model3 \| \| \| --- \| --- \| --- \| --- \| --- \| --- \| --- \| --- \| --- \| --- \| --- \| \| OR (95%CI) \| *P* \| OR (95%CI) \| *P* \| OR (95%CI) \| *P* \| \| Treatment protocol \| 62.42 \| 0.587 \|  \|  \|  \|  \|  \|  \|  \|  \| \| 1 \| 67.65 \| 1.00 (Reference) \| 1.00 (Reference) \|  \|  \| 1.00 (Reference) \|  \|  \| 1.00 (Reference) \|  \| \| 2 \| 61.90 \| 0.690 \| 1.29 (0.58 ~ 2.84) \| 0.533 \|  \| 1.52 (0.63 ~ 3.69) \| 0.354 \|  \| 1.60 (0.64 ~ 4.00) \| 0.319 \| \| 3 \| 50.00 \| 0.518 \| 0.70 (0.17 ~ 2.88) \| 0.621 \|  \| 0.47 (0.10 ~ 2.12) \| 0.324 \|  \| 0.38 (0.08 ~ 1.85) \| 0.232 \|   Treatment protocol：1. 375 mg/m² Protocol 2. B-cell Level-Driven Protocol3. Other Low-Dose Protocol  Model1: Crude |
| --- | --- | --- | --- | --- | --- | --- | --- | --- | --- | --- | --- | --- | --- | --- | --- | --- | --- | --- | --- | --- | --- | --- | --- | --- | --- | --- | --- | --- | --- | --- | --- | --- | --- | --- | --- | --- | --- | --- | --- | --- | --- | --- | --- | --- | --- | --- | --- | --- | --- | --- | --- | --- | --- | --- | --- | --- | --- | --- | --- | --- | --- |
| Model2: Model2: Adjust: gender, age, BMI, Hypertension, Diabetes, Infection, Total cholesterol (TC), Triglycerides (TG), LDL |
| Model3: Adjust: gender, age, BMI, Hypertension, Diabetes, Infection, Total cholesterol (TC), Triglycerides (TG), LDL, Hormone use, ACEI use  **Supplementary Table 2 Effects of different rituximab regimens on 12-month outcomes (crude to multivariable-adjusted analyses)**   \| Variables \| Remission (%) \| P \| Model1 \| \|  \| Model2 \| \|  \| Model3 \| \| \| --- \| --- \| --- \| --- \| --- \| --- \| --- \| --- \| --- \| --- \| --- \| \|  \|  \| OR (95%CI) \| *P* \| OR (95%CI) \| *P* \| OR (95%CI) \| *P* \| \| Treatment protocol \| 70.47 \| 0.365 \|  \|  \|  \|  \|  \|  \|  \|  \| \| 1 \| 79.41 \| 1.00 (Reference) \| 1.00 (Reference) \|  \|  \| 1.00 (Reference) \|  \|  \| 1.00 (Reference) \|  \| \| 2 \| 68.57 \| 0.319 \| 0.82 (0.34 ~ 1.96) \| 0.657 \|  \| 0.97 (0.37 ~ 2.57) \| 0.959 \|  \| 0.97 (0.35 ~ 2.63) \| 0.945 \| \| 3 \| 60.00 \| 0.406 \| 0.84 (0.18 ~ 3.97) \| 0.826 \|  \| 0.50 (0.10 ~ 2.59) \| 0.408 \|  \| 0.39 (0.07 ~ 2.19) \| 0.283 \|   Treatment protocol：1. 375 mg/m² Protocol 2. B-cell Level-Driven Protocol3. Other Low-Dose Protocol  Model1: Crude  Model2: Model2: Adjust: gender, age, BMI, Hypertension, Diabetes, Infection, Total cholesterol (TC), Triglycerides (TG), LDL  Model3: Adjust: gender, age, BMI, Hypertension, Diabetes, Infection, Total cholesterol (TC), Triglycerides (TG), LDL, Hormone use, ACEI use |
|  |
